# Multidisciplinary outpatient care program for patients with chronic low back pain: design of a randomized controlled trial and cost-effectiveness study [ISRCTN28478651]

**DOI:** 10.1186/1471-2458-7-254

**Published:** 2007-09-20

**Authors:** Ludeke C Lambeek, Johannes R Anema, Barend J van Royen, Peter C Buijs, Paul I Wuisman, Maurits W van Tulder, Willem van Mechelen

**Affiliations:** 1 Department of Public and Occupational Health VU University Medical Center, Amsterdam, The Netherlands; 2 Institute for Research in Extramural Medicine, VU University Medical Center, Amsterdam, The Netherlands; 3 Body@Work, Research Center Physical Activity, Work and Health, TNO-VU-University Medical Center, Amsterdam, The Netherlands; 4 Research Center for Insurance Medicine AMC-UWV-VUmc, Amsterdam, The Netherlands; 5 Institute for Health Sciences, Faculty of Earth & Life Sciences, Vrije Universiteit, Amsterdam, The Netherlands; 6 Department of Orthopaedic Surgery, VU University Medical Center, Amsterdam, The Netherlands

## Abstract

**Background:**

Chronic low back pain (LBP) is a major public and occupational health problem, which is associated with very high costs. Although medical costs for chronic LBP are high, most costs are related to productivity losses due to sick leave. In general, the prognosis for return to work (RTW) is good but a minority of patients will be absent long-term from work. Research shows that work related problems are associated with an increase in seeking medical care and sick leave. Usual medical care of patients is however, not specifically aimed at RTW.

The objective is to present the design of a randomized controlled trial, i.e. the BRIDGE-study, evaluating the effectiveness in improving RTW and cost-effectiveness of a multidisciplinary outpatient care program situated in both primary and outpatient care setting compared with usual clinical medical care for patients with chronic LBP.

**Methods/Design:**

The design is a randomized controlled trial with an economic evaluation alongside. The study population consists of patients with chronic LBP who are completely or partially sick listed and visit an outpatient clinic of one of the participating hospitals in Amsterdam (the Netherlands). Two interventions will be compared. 1. a multidisciplinary outpatient care program consisting of a workplace intervention based on participatory ergonomics, and a graded activity program using cognitive behavioural principles. 2. usual care provided by the medical specialist, the occupational physician, the patient's general practitioner and allied health professionals. The primary outcome measure is sick leave duration until full RTW. Sick leave duration is measured monthly by self-report during one year. Data on sick leave during one-year follow-up are also requested form the employers. Secondary outcome measures are pain intensity, functional status, pain coping, patient satisfaction and quality of life. Outcome measures are assessed before randomization and 3, 6, and 12 months later. All statistical analysis will be performed according to the intension-to-treat principle.

**Discussion:**

Usual care of primary and outpatient health services isn't directly aimed at RTW, therefor it is desirable to look for care which is aimed at RTW. Research shows that several occupational interventions in primary care are aimed at RTW. They have shown a significant reduction of sick leave for employee with LBP. If a comparable reduction of sick leave duration of patients with chronic LBP of who attend an outpatient clinic can be achieved, such reductions will be obviously substantial for the Netherlands and will have a considerable impact.

**Trial registration:**

ISRCTN28478651

## Background

### Primary care in the Netherlands

In the Netherlands primary care for patients with low back pain (LBP) is given by general practitioners, occupational physicians, and allied health professionals e.g. physical therapists or occupational therapists. Usually a patient first visit a general practitioner. The general practitioner may treat the patient himself or refer the patient to a medical specialist in outpatient care and/or to an allied health professional in primary care. Since January 2006, patients do also have direct access to allied health professionals.

General practitioners and physical therapists have their own national clinical guidelines for LBP [1,2]. Medical specialists do not have clinical guidelines for LBP, although a multidisciplinary guideline has been developed and published in 2004 [3]. Usual care is assumed to be consistent with guideline recommendations.

Each Dutch company is obliged to have company insurance for sick leave and to offer their employees access to occupational health care. Occupational physicians provide social medical guidance for sick listed employees with the aim to return to work (RTW) as quickly as possible. Self-employed people, however, don't have access to occupational health care. They need to have a private insurance for incapacity for work, which is expensive. If they have insurance a physician working for an insurance company will guide the self-employed individual back to work. Occupational physicians also have their own national clinical guidelines [4].

### Low back pain

LBP is a major public and occupational health problem and associated with very high costs. The total annual cost of low back pain to Dutch society, is estimated to be € 4.6 billion per year. The economic burden of LBP is primarily related to indirect costs (93%) of productivity losses due to sick leave and long-term disability. The direct healthcare costs are much lower (7%) [5]. In general, the prognosis for RTW is good [6]. However, approximately 10% to 25% of patients will have long-term absenteeism from work and will be at risk of social and financial deprivation. These patients account for 75% of the indirect costs of LBP [7].

Research shows that work-related problems are associated with an increase in seeking medical care and sick leave [8]. Usual care of medical specialists, general practitioners and allied health professionals of patients sick listed due to LBP is not directly aimed at RTW and co-operation with occupational physicians is usually poor [9]. Besides the content of the usual care, also the limited information exchange between the treating and occupational physicians is often an obstacle for RTW [10,11]. Despite these findings, several studies indicate that medical specialists, general practitioners and occupational physicians are willing to improve cooperation and communication [12,13].

Because usual care of primary and outpatient health services isn't directly aimed at RTW, it is desirable to look for care which is aimed at RTW. Research shows that several occupational interventions in primary care are aimed at RTW. They have shown a significant reduction of sick leave for employee with LBP [14,15].

### Objectives

In this article, we describe the design of a randomized controlled trial (RCT) comparing multidisciplinary outpatient care with usual clinical medical care for patients with chronic LBP visiting an outpatient clinic. The multidisciplinary outpatient care program consists of two interventions: a workplace intervention and a graded activity intervention. This program will be compared to usual clinical medical care. The first question of this study is 'Is the multidisciplinary outpatient care situated in both primary and outpatient care setting for patients with chronic LBP effective for RTW and cost-effective compare to the usual clinical medical care?'. The second question is 'How is the program for multidisciplinary outpatient care and its implementation (i.e. the applicability, compliance to, perceived effectiveness, barriers) evaluated by patients with LBP, their employer and their health care professionals?'

## Methods/Design

### Study design

#### Organization of the study

The design of the study is an randomized controlled trial (RCT) with a full economic evaluation alongside. The conduct of the study is guided by a committee of representatives of all professional groups monitoring the implementation of the interventions in the study. The most important task of this committee was the critical appraisal of the study protocol on the feasibility of the interventions during the study.

**Figure 1:**
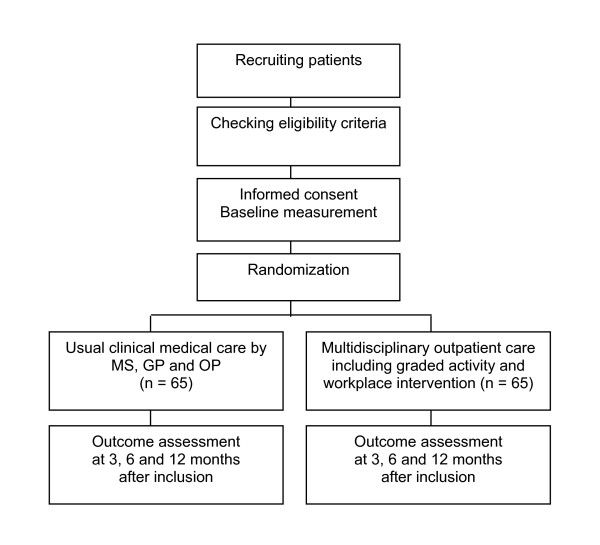
**Design of the RCT**. MS, medical specialist; GP, general practitioner; OP, occupational physician.

The Medical Ethics Committees of the participating hospitals (the VU University Medical Centre, the Slotervaart hospital, the Amstelland hospital, the Onze Lieve Vrouwe Gasthuis, all based in Amsterdam, and the Spaarne hospital in Hoofddorp) approved the study. All participants will sign an informed consent and will be insured according to Dutch Law in case of any damage caused by participation in the study. Figure 1 shows a brief outline of the design of the RCT.

#### Participants

The population consists of LBP patients (18–65 years) who visit an outpatient clinic (mainly orthopaedics and neurology, but also rheumatology and neurosurgery) of one of the participating hospitals. The population has to 1. have LBP lasting more than 12 weeks, 2. have paid work (i.e. paid-employment or self-employed) for at least 8 hours a week and 3. be on (partially) sick leave. Patients will be excluded from the study in case of 1) being sick listed more than two years; 2) working temporarily for an employment agency without detachment; 3) having specific low back pain, due to infection, tumor, osteoporosis, rheumatoid arthritis, fracture or inflammatory process; 4) having had a lumbar spine surgery in the last 6 weeks or having to undergo surgery or invasive examinations in the near future (within 3 months); 5) having serious psychiatric disorders; 6) having cardiovascular or medical contraindications for physical activity according to the Physical Activities Readiness Questionnaire (PAR-Q) [16]; 7) being pregnant or having given birth in the last 3 months; 8) dealing with a lawsuit to their employer; 9) not having the ability to complete questionnaires written in the Dutch language.

In order to recruit a sufficient number of eligible patients, in each hospital a competent hospital employee will identify the source population weekly from the computerized patient record system. Within one week after the visit, the patients will receive three documents by mail: 1. a letter from their medical specialist in which they are informed about the study and asked if they are willing to participate, 2. a brochure with details of the study and 3. a short screening questionnaire. A return envelope is enclosed for returning the screening questionnaire to the researcher. On this questionnaire they can indicate if they are willing to participate in the study.

All patients who return the questionnaire, meet the criteria and indicate that they are willing to participate will be contacted by telephone. The researcher or a research assistant will provide additional information about the implications of participation and will check the eligibility of the patient. If a patient meets the selection criteria and is willing to participate, they will be asked to sign the informed consent.

#### Randomization

An independent statistic performs randomization, using a computer-generated random-sequence table. The randomization will be performed on patient level. The patients are stratified on 2 important prognostic factors before randomization to prevent an unequal distribution. The first prognostic factor is the duration of sick leave (less than or longer than 3 months). The second prognostic factor is job characteristics ((mainly) physical or mental work demands) [17]. This results in a total of 4 strata. For every stratum, block randomization of 4 allocations will be used to avoid unequal treatment group sizes.

The researcher will prepare for each stratum opaque, sequentially numbered, and sealed coded envelopes, with either a note for the multidisciplinary outpatient care group or a note for the UC group. If a patient meets all criteria he/she will be allocated to one of the four strata. The researcher hands over to the patient the first two envelopes (left over) of that stratum where the patient is asked to pick one of the envelopes, open the envelope and sign the note. After randomization, the researcher/assistant registers information in the database about the patient's employer, occupational physician, general practitioner, and medical specialist. Patients in the multidisciplinary outpatient care group choose one of the 10 physiotherapy practices, in which they will perform the graded activity program. After randomization, the researcher/assistant will make, for patients in the multidisciplinary outpatient care group, an appointment for a visit with the care manager of the multidisciplinary outpatient care program. This appointment will take place within one week after randomization.

#### Interventions

##### 1. Multidisciplinary outpatient care program (MOC)

**Figure 2:**
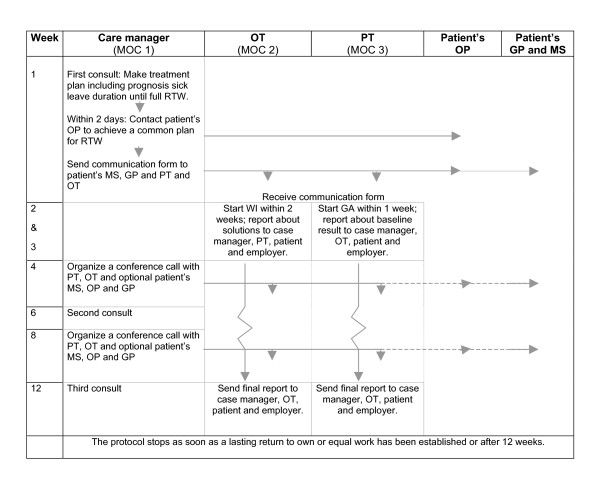
**Time schedule of the multidisciplinary outpatient care protocol**. GA, graded activity; GP, general practitioner; OT, occupational therapist; MS, medical specialist; OP, occupational physician; WI, workplace intervention; PT, physicial therapist; RTW, return to work.

Multidisciplinary outpatient care is a case management program consisting of a workplace intervention protocol and a graded activity (GA) program. Two clinical occupational physicians were trained as care managers. The content of the interventions and the role of each member of the multidisciplinary team are described in detail below. The flow of the multidisciplinary outpatient care program is shown in Figure 2.

##### MOC 1: Case management protocol

The multidisciplinary team providing the multidisciplinary outpatient care consists of the care manager, the occupational therapist, the physical therapist and the patients own medical specialist, general practitioner and occupational physician. The care manager has an intermediate role between primary and outpatient care. He is responsible for the planning and the coordination of care and for communication with the other care providers involved in the team.

The patient will visit the care manager within 1 week after randomization. The care manager starts with history taking and physical examination. History taking aims to identify functional limitations at work and factors that could be of influence for RTW, such as heavy work, organizational problems or inadequate treatment [4]. If the care manager has doubts about the clinical diagnosis or functional limitations of the patient, he contacts the patient's medical specialist, occupational physician or general physician. By the end of the first consultation, the care manager proposes a treatment plan, gives an estimate of the sick leave duration until full RTW and gives the patient a file for collecting all documents of the multidisciplinary team. If the patient agrees with the plan, the care manager will contact the patient's occupational physician in order to advise the plan for RTW. The final advise to the patient and his/her employer with respect to RTW will remain the responsibility of the patient's occupational physician. When the patient's occupational physician agrees with the plan, the care manager sends the communication form to the medical specialist, general practitioner, physical therapist and occupational therapist. All communication will be performed according to the GP-OP-coordination guideline from the Dutch general practitioner and occupational physician medical organizations [4,11]. The communication form contains information about the history taking and physical examination, personal information of the patient and a prognosis for the sick leave duration until full RTW.

To streamline the process, the care manager has access to an administrative database, which contains information about the health care professionals involved, the employer of patients and generates the tasks of the care manager per patient on a daily basis. One of the tasks is to organize a conference call every three weeks. A conference call will take place between the care manager, occupational therapist en physical therapist and the patient's medical specialist, general practitioner and occupational physician if relevant. After 6 and 12 weeks, the patient will visit the care manager again to evaluate progress and if necessary adjust the date of RTW.

##### MOC 2: Workplace intervention protocol

The workplace intervention consists of work(place) adaptations and is based on active participation and strong commitment of both the patient and employer. The workplace intervention is based on methods used in 'participatory ergonomics' [18-20]. Prior the start of the BRIDGE-study, three occupational therapists were trained by an expert to provide the workplace intervention protocol.

The process of the workplace intervention protocol is described in Table 1. The total duration of the intervention is eight hours, within 4 weeks. For each patient a team is formed that includes the occupational therapist (process mediator), the patient, the patient's supervisor, and other potential stakeholders (e.g. a Human Resources manager). Even in case the company itself has adjusted a workplace earlier, the occupational therapist makes an inventory of the workplace and the patient's tasks and informs the physical therapist and the care manager.

**Table 1:** Steps of the workplace intervention protocol

**Step**	
0	Within 2 weeks after the patient visit the care manager, the OT makes by telephone an appointment with patient and patient's supervisor for the first visit of the workplace intervention protocol.
1	First visit consists of:
	1 Patient's workplace observation and inventory and ranking patient's tasks and obstacles for RTW by the patient.
	2 Inventory and ranking patient's tasks and obstacles for RTW by the patient's supervisor
	3 Patient, patient's supervisor and the OT brainstorm and discuss about as many solutions as possible to clear the obstacles for RTW.
2	Within two days after the OT has visited the workplace, the OT reports about all solutions and actions in a report to the patient, the patient's supervisor and the multidisciplinary team.
3	An optional worksite visit to give additional instructions or training to the patient will take place if necessary. The moment of execution depends on whether adjustments on the worksite have to be made first.
4	Four weeks after the first visit, an evaluation by telephone will take place between the patient and the OT with regard to the implementation of the solutions agreed upon. If necessary, a stakeholder has to be found for further support of improvements.
5	Within two days after the telephone evaluation, a final report is sent to the multidisciplinary team to report the progress of the protocol.

OT, occupational therapist; RTW, return to work.

The aim of the workplace intervention is to achieve consensus between patient and supervisor regarding feasible solutions for the obstacles for RTW. The solutions are judged on availability, feasibility and solving capability. After consensus, the occupational therapist, patient, patient's supervisor and potential other stakeholders agree on a plan of action. Responsibility for implementing the plan of action is put on the patient's and supervisor's account as much as possible.

##### MOC 3: The Graded Activity Program (GA)

This program is based on the principles of graded activity (GA) as developed by Lindström et al. [21,22] and on the operant (conditioning) model described by Fordyce [23]. It is adjusted to the Dutch situation in the study of Staal et al. 2004 [15]. The program used in this study has been adjusted to the particular study population of the BRIDGE-study. This GA program is provided by 10 regional physiotherapy practices located in Amsterdam or in the surrounding area. These practices have already been trained in the GA program in the context of other research projects [15,24,25]. Prior to the study, all physical therapist have followed a practical training course in the GA program (given by trainers) and were updated on the content of the BRIDGE-study.

The aim of the GA program is restoring occupational function and returning to previous work. Its aim is not pain reduction. The essence of the GA program is to implement an individually graded exercise program, which teaches the patient that it is safe to move while increasing the level of activity. During the GA program the patient has an active role in RTW and the physical therapist acts as a coach and supervisor, using a hands-off approach.

The GA program has two phases: 1. a baseline phase (pain/contingent); 2. a treatment phase (time/contingent). In Table 2 the content of the protocol is described. The entire program consists of maximally 26 sessions. The three baseline sessions have to be done in one week. The maximum duration is 12 weeks. However, the program stops as soon as a lasting return to own or equal work has been established. The session limit will not be communicated to the patient, because it will probably lead to a time lag regarding RTW.

**Table 2:** Steps of the graded activity protocol

**Step**	**Characteristics**	
		The GA program starts within one week after the patient visits the care manager.

Before starting the baseline phase		-The PT performs a history-taking and a physical examination; -The PT gives counseling using the bio-psycho-social model on the development and maintenance and the consequences of pain; -The PT states that it is safe to start the GA program.

**1 Baseline phase**	**Pain Contingent** Patient may stop at any time if he feels pain or other discomfort Individual training 3 sessions of 1.5 hour	- Baseline consists of 6 fixed and 3 free exercises (simulating work situation); - Aim is to determine the maximal performance for each exercise separately; - According to the results, a start quota (70% of the mean) and the load of each exercise session until the end of the program will be set; - The load of each quota depends on the date of full RTW; - When all quota are set, the PT will send the baseline results and the treatment report to the employee, the employer and the multidisciplinary team.

**2 Treatment phase**	**Time contingent** Pain is not a reason for stopping or altering the program Group training twice a week (4–6 persons) 1 hour per session	- The pre-set quota have to be followed strictly; - The PT accompanies the treatment sessions and evaluates the sessions; - A positive reinforcement will be given by the PT after completion of the quota; - When the date of RTW is within a few weeks, the learned behaviour and management of pain will be discussed. At this point the frequency of the sessions will be decreased to once a week until the patient returns to work; - When the employee fully returns to work or after 26 sessions the protocol will stop. A final communication form will be send to the multidisciplinary team.

		Every three weeks, the PT sends an evaluation form to the multidisciplinary team to report the progress of the treatment protocol.

		When the patient is sick listed because of LBP within 4 weeks after RTW, the GA program will be continued. It will again stop as soon as the patient fully returns to work, or all 26 sessions of the GA program are given.

GA, graded activity; PT, physical therapist; RTW, return to work; LBP, low back pain.

##### 2. Usual clinical medical care (UC)

The patients who were allocated to the UC group receive the usual guidance by their medical specialist, occupational physician and general practitioner and allied health professionals. There are no specific requirements or restrictions with regard to type, duration or frequency of treatment. The patient's own general practitioner, occupational physician and treating medical specialist will be informed by letter about the study and the allocation of their patient to the UC group. They will be asked to adhere to their professional guidelines for LBP (if available) [1,3,4].

### Outcome assessment and data-collection

#### 1. Quantitative outcome assessment

Most of the outcome variables, are reported by means of self-reported questionnaires and will be assessed four times by all participants: before randomization (T0), at the end of 3 months (T1), at the end of 6 months (T2) and 12 months after randomization (T4). Prognostic outcomes are assessed at T = 0. The direct and indirect medical costs are measured four times by means of cost diaries. This cost diary is inserted into the questionnaire at 3, 6, and 12 months after randomization and is sent at 9 months after randomization separately to the participant. For the outcome RTW, sick leave data are gathered by mailing a calendar monthly and are checked with the data records of the occupational health services after 12 months. Patients are reminded by telephone after two weeks if they have not returned the questionnaire, the cost diaries or the calendar yet. Table 3 presents the outcome variables, the used instruments and the timing of the data collection.

**Table 3:** Overview of variables measured in this study

Variable	Time Measured				
	Baseline T0	3 months	6 months	9 months	12 months
		T1	T2	T3	T4

Inclusion/exclusion	x				
Informed consent	x				
Randomization	x				
Prognostic variable					
Demographic variables (age, gender, ect)	x				
Potentional work-related physical factors (DMQ)	x				
Potential work-related psychosocial factors (JCQ)	x				
Outcome measures					
Primary					
Return to work	x	x	x		x
Secondary					
Pain intensity (VAS)	x	x	x		x
Functional status (RDQ-24)	x	x	x		x
Pain coping (PCI)	x	x	x		x
Quality of life (Euroqol)	x	x	x		x
Patient Satisfaction with Occupational Health Services (PSOHSQ)		x			
Cost diaries		x	x	x	x

#### 2. Qualitative outcome assessment

Outcome variables related to the implementation of the protocol will be gathered by 1. additional questions about the multidisciplinary outpatient care program for patients in the multidisciplinary outpatient care (MOC) group, which are inserted into the questionnaire at T1; 2. an in-depth interview about the multidisciplinary outpatient care program with the first 30 patients randomized to the MOC group, which is done by telephone at T1. The interview will take approximately one hour; 3. questionnaires, sent to the stakeholders involved (employer/supervisor, patient's own GP and OP, professionals of the multidisciplinary team) at T1; 4. two focus groups for 5 to 7 professionals of the multidisciplinary team (care manager, physical therapist, occupational therapist and medical specialist) organized during the first year of recruitment of patients.

### Outcome measures

#### Primary outcome

The primary outcome is RTW defined as: "duration of sick leave in calendar days from the day of randomization until full RTW in own or other work with equal earnings, for at least 4 weeks without (partial or full) recurrence. In addition, the total duration of sick leave due to LBP (including all recurrences of sick leave episodes due to LBP) will be calculated for the entire follow-up period. Patients are asked monthly 1. to register the percentage of sick leave i.e. dividing the number of hours a patient is sick listed by the number of hours of the patients working week multiplying with a 100%; 2. the reason for being on sick leave, i.e. LBP, influenza ect. After a patient has been followed for a period of 12 months, a letter will be sent to the patient's occupational physician with the request to provide the sick leave data of the last 12 months registered in the data records of the occupational health services. Because some patients are self-employed, the self-reported data will be used for statistics.

#### Secondary outcomes

- Pain intensity is measured by three short questions using a 10-point Visual Analogue Scale (VAS) [26].

- Functional status is assessed by the Roland-Morris Disability-24 Questionnaire (RDQ-24), which has shown to be useful in LBP research [27,28].

- Pain coping is measured with the Pain Coping Inventory Scale (PCI) [29].

- Quality of life is measured with the Dutch translation of the Euroqol instrument [30].

- Cost diaries are used to measure direct (non)-medical costs. The cost diary includes direct health care costs relevant to the treatment of LBP, such as visits to a general practitioner, occupational physician, manual therapist, physical therapist, other exercise therapy or complementary health therapists (e.g. acupuncturist), visits to a medical specialist in orthopaedic surgery, neurology, rheumatology, or rehabilitation medicine, and hospitalization. Direct non-health care costs include homecare and costs for paid and unpaid help due to the disability.

- A process evaluation will be conducted after implementation of the protocol and inclusion of the first 40 cases treated with the multidisciplinary outpatient care program. Quantitative and qualitative data about the applicability, compliance, satisfaction and barriers related to the (implementation of the) protocol will be collected. Patient satisfaction will be measured with the Patient Satisfaction with Occupational Health Services Questionnaire (PSOHQ)[31].

#### Prognostic measures

- Potential work-related psychosocial factors is measured with the job content questionnaire (JCQ)[32].

- Data on workload is measured with the Dutch Musculoskeletal Questionnaire (DMQ)[33].

### Sample size

We assume that a Hazard Ratio (HR) of 2.0 indicates a relevant difference between the multidisciplinary outpatient care group and the UC group. This HR is based on HRs found in comparable studies in primary care [15,19,34]. Another assumption is that 40 percent of the sick listed patients due to chronic low back pain does not return to work during the follow-up period (12 months after randomization). We expect a dropout rate of 10%, based on experiences with comparable research [35,36]. To get a complete data set of 115 patients, 130 patients with chronic LBP who are sick listed will be included (HR = 2, with a power (1-β) of 80% and a significance level of 5%) [37]. Before starting the inclusion of patients, a two-week visitors' waiting room survey has been done at the departments of orthopaedics and neurology at the VU Medical Center Amsterdam. This survey showed that at these two departments the percentage of patients sick listed because of LBP is low (3–4%). Because of this low percentage, four other hospitals are recruited so the recruitment period of 24 months duration is likely acceptable.

### Blinding

Patients, therapists and researchers cannot be blinded for the allocated treatment. Treatment allocation takes place after informed consent and completion of the baseline questionnaire. Most outcome measures are self-reported and consequently cannot be blinded. However, since all questionnaires are sent to the patient by mail, researcher and care providers are not likely to influence the way patients complete the questionnaires. In addition, the reliability regarding the self-reported data on RTW is checked by sick leave data derived from databases of occupational health services. The therapists of the multidisciplinary team (care manager, occupational therapist, physical therapist) are not involved in assessing any of the outcomes.

After randomization all patients receive their own code. A run-up number and the first two letters of the visited hospital compose this code. The research assistant will put all data in the computer by code. Therefore, the analysis of the data by the researcher will be blind.

### Co-interventions and compliance

During the intervention period co-interventions are discouraged, but cannot always be avoided. Asking patients and therapists independently about all interventions applied will assess the compliance to the multidisciplinary outpatient care program. Information about all treatments and co-interventions received by the patients in the multidisciplinary outpatient care and UC group, are collected by means of cost-diaries and questionnaires.

### Data analysis

All analyses will be performed at patient level. To examine the success of randomization, descriptive statistics will be used to compare the baseline measurements of the two groups. If necessary, analyses will be adjusted for prognostic dissimilarities. The primary independent variable in the analyses will be the treatment (MOC or UC) to which the patient is allocated. The primary dependent variable is sick leave duration in days until full return to own or equal work. Kaplan Meier analyses (including log rank test) will be used to describe the univariate association between group allocation and the sick leave duration until full RTW. The Cox Proportional hazard model will be used to analyze the HR of the RTW rates in both groups in a multivariate model. Longitudinal random coefficient analyses will be used to assess differences between treatment groups in improvement in all secondary outcome measures. The baseline value of the particular outcome variable will be added to the model in order to correct for possible regression to the mean.

All statistical analyses will be performed according to the intention-to-treat principle, i.e. the patients will remain in the group to which they were randomly allocated at baseline. In order to assess whether protocol deviations have caused bias, the results of the intention-to-treat analyses will be compared to per-protocol analyses. Only those patients who complied fully with the intervention protocol will be included.

### Economic evaluation

Cost-effectiveness will be evaluated from the societal perspective including both direct and indirect costs. Both costs groups will be estimated according to the Dutch guidelines for cost analysis in health care research [38,39] or with the use of the tariffs of the Dutch Central Organization for Health Care Charges [40]. These costs will be measured 4 times during the one-year follow-up. Indirect costs of loss of production due to LBP are not related to health care, but are costs in paid and unpaid labour as a consequence of sickness, sick leave, disability and/or death of a productive person. Mailing the patient monthly a calendar as well as collecting the sick leave data collected in occupational health services database will measure the number of days on sick leave due to LBP. Quality of life will be measured according to the standard Dutch version of the EuroQol [30]. Costs will be summated for each individual patient. Bootstrapping will be used for pair-wise comparison of the mean groups to calculate mean differences in direct, indirect and total costs between the two groups of patients. Confidence intervals (95%) will be obtained by bias corrected and accelerated bootstrapping. A cost-effectiveness ratio will be calculated by dividing the difference between the mean costs of the intervention groups by the difference in RTW between the intervention groups. A cost-utility analysis will also be conducted in which the incremental costs per QALY will be estimated. Reliability of the cost-effectiveness ratios will be graphically presented on a cost-effectiveness plane and acceptability curves.

## Discussion

This study addresses an important question because LBP is a major health and economic problem. In the BRIDGE-study we focus on patients, sick listed because of chronic LBP, who are seeking for outpatient care by consulting a medical specialist. These patients frequently have problems with their working capacity [8]. However, there is strong evidence that most clinical interventions for chronic LBP are not effective for RTW. Therefore, a protocol is developed to evaluate the clinical and cost effectiveness of a multidisciplinary outpatient care program situated in both primary and outpatient care for patients with chronic LBP attending a medical specialist. The multidisciplinary outpatient care program consists of a case management protocol including a workplace intervention protocol and a GA program. Recent research shows that long-term work disability is not only due to patient's personal characteristics but is also a consequence of an interaction with the patient's environment (the workplace system, compensation system, healthcare system) [41]. In this study the GA is directed to the patient's personal characteristics, and illness behavior (cognitions, coping) via operant conditioning, the workplace intervention will focus on the work system (worker, supervisor) while the coordination of care of the care manager will be directed to focus on the health care system to a common RTW-goal.

### Strengths and limitations of this study

The main focus of this study is RTW which we will measure both monthly by calendar for one year as well as check against data records of the occupational health services after the follow-up period ends. This will avoid information bias for our primary outcome.

Another strength of this study is that, besides a quantitative analysis, a qualitative analysis will be done as well. Data for the qualitative analysis are gathered by focus groups and an in-depth interview. We have chosen for organizing focus groups because it has several advantages. First, it provides insight in the sources of complex behaviour and motivations. It also gives better insight on consensus and disagreement between the participants around topics. The added value of an in-depth interview is that the patients can tell about their experiences with the multidisciplinary outpatient care program in a more comprehensive way then they can do in the questionnaires. Moreover the patients can discuss topics in the depth interview, which the questionnaire will not been offer them. With all these data it is possible to identify aspects of the working mechanism (black box) behind the multidisciplinary outpatient care program. It will be possible to clarify the elements that contribute to the effects of the multidisciplinary outpatient care program, which results in more valid conclusions.

There are also some limitations. First, blinding is a validity criterion in most reviews, which is not possible in this trial. It is impossible to blind patients and healthcare providers due to the character of the workplace intervention and the GA program. Another possible source of bias is the difference in attention patients might receive, the so-called 'Hawthorne effect'. Patient in the multidisciplinary outpatient care group will receive more attention than the patient in the UC group. This might lead to overestimation of the effect of the multidisciplinary outpatient care program.

Our economic evaluation will be performed from the societal perspective. It would be desirable to do additional analysis from the employer's perspective, since interventions in occupational care in the Netherlands have to be paid by employers. However, the population of this study is working for different companies, which are connected to different occupational health care providers or is self-employed. Thus, gathering all the information needed for the economic evaluation is impossible.

### Impact on this study

It has been shown in a primary care setting that occupational interventions resulted in a reduction in sick leave of 29–105 days, depending on the occupational intervention applied and the population [15,42]. This equals an average reduction of sick leave duration of 33%. If a comparable reduction of sick leave duration of patients with chronic LBP of who attend outpatient clinics can be achieved, such cost reductions will be obviously substantial for the Netherlands. Therefore, to improve RTW of patients with chronic LBP in outpatient care and to reduce costs, multidisciplinary outpatient care can have a considerable impact.

## Abbreviations

GA, graded activity; GP, general practitioner; HR, hazard ratio; LBP, low back pain; MOC, multidisciplinary outpatient care; OP, occupational physician; Par-Q, Physical Activities Readiness Questionnaire; PCI, Pain Coping Inventory scale; RCQ-24, Roland – Morris Disability-24 Questionnaire; RCT, randomized controlled trial; RTW, return to work; UC, usual clinical medical care; VAS, Visual Analogue Scale.

## Competing interests

The author(s) declare that they have no competing interests.

## Authors' contributions

All authors have been involved in the development of the study design and research protocols. LCL and JRA participated in the general coordination of the study and drafted the manuscript. LCL carried out data collection. All authors read and corrected draft versions of the manuscript and approved the final manuscript.

## Pre-publication history

The pre-publication history for this paper can be accessed here:


